# The thermo-fluid and exergy analysis of a shell and twisted helical tube heat exchanger

**DOI:** 10.1038/s41598-026-56961-0

**Published:** 2026-06-26

**Authors:** Mahmoud Abdelmagied

**Affiliations:** Refrigeration and A/C Technology Department, Faculty of Technology and Education, Capital University (Formerly Helwan), Cairo, 11282 Egypt

**Keywords:** Twisted helical tubes, Coil diameters, Shell and tube, Exergy, Energy science and technology, Engineering

## Abstract

The present study experimentally investigates a shell and twisted helical tube heat exchanger (*STHTHX*), which employs a twisted helical tube configuration to enhance thermofluid performance under turbulent flow conditions. The effects of the twisted helical coil diameter (*D*), inner Reynolds number (*Re*_*i*_), and shell Reynolds number (*Re*_*sh*_) on the thermal performance (*η*) and exergy characteristics of the *STHTHX* are examined and compared with those of a conventional shell and smooth helical tube heat exchanger (*SSHTHX*). The twisted helical tube design is expected to intensify the swirl motion of the fluid and induce additional flow disturbances and improve the thermofluid characteristics. Three test specimens were fabricated and tested with twisted coil diameters (*D*) of 0.095, 0.125, and 0.165 m. The experiments were carried out under counter-flow conditions with inner mass flow rates (*ṁ*_*i*_) ranging from 0.033 to 0.181 kg/s, corresponding to Reynolds numbers (*Re*_*i*_) from 6.1 × 10^3^ to 4.4 × 10^4^ in the twisted helical coil, and shell-side mass flow rates (*ṁ*_*sh*_) between 0.067 and 0.175 kg/s. This study aims to fill the research gap regarding the thermofluid characteristics and exergy analysis of the *STHTHX* and to provide experimental guidance for its design and optimization. The results reveal that the *STHTHX* demonstrated superior thermofluid performance compared to *SSHTHX*. Reducing the twisted coil diameter from 0.165 to 0.095 m increasing $\overline{{Nu_{i} }}$, and *f*_*i*_ by 47.9, and 38.77%, respectively. The *STHTHX* achieved a maximum *η* of 2.07 at *D* = 0.095 m. New correlations are proposed to predict $\overline{{Nu_{i} }}$ and *f*_*i*_.

## Introduction

The global energy demand’s steep rise and the finite fossil fuel supplies have prompted the search for alternative energy sources and energy systems. Additionally, the energy sources that are accessible must be utilized effectively. In light of that, several studies are conducted to improve current energy conversion technologies and create new systems. The system’s thermal efficiency is directly affected by the heat exchangers’ performance, which are commonly used to transfer the heat from one energy source to another. Among the heat exchanger configurations, the shell and twisted helical tube heat exchanger is commonly utilized. The twisted helical tube within the shell serves as the primary design element of this exchanger, owing to its compact design and superior thermal effectiveness. The curvature of the helical tube induces centrifugal forces and a secondary flow inside the tubes, which enhances fluid mixing, significantly increases heat transfer rates, and consequently improves overall performance^1^. Furthermore, compared to straight-tube configurations, the compact design of the twisted helical coil occupies less space while providing high thermal stress flexibility—an essential feature for various industrial applications such as heat recovery, food industries, air-conditioning, cold and frozen storage systems, refrigeration units, chemical processes, thermal power plants, and water desalination systems^2,3^. The design of the *STHTHX* is one of the passive enhancement techniques due to the presence of the tube twist. It is anticipated that this improved profile would improve heat transmission. Because of the increased turbulence and flow fluctuation created in the fluid route, twisted tube and helical coil combinations should improve heat transfer more effectively. Forming a twisted tube from a circular, straight tube is simple. A circular tube is marked on the lathe at one end, and the other end is cold drawn with a revolving die on the outside, creating internal twisted grooves that resemble the exterior groove^4^.

Abdelmoety et al.^5^ investigate numerically the heat transfer performance, pressure drop, friction factor, exergy, and performance enhancement requirements of a heat exchanger caused by a geometrical alteration on a cylindrical shell of a U-tube design. The suggested design improved the thermal performance and the cost. Addis et al.^6^ presented numerically the thermo-hydraulic performance of parallel multi-shell and multi-helical tubes. These results show that the enhancement ratio of *U*_*o*_ is larger in a design with two helical tubes than in a configuration with three helical tubes. Biswal et al.^7^ investigates numerically the unsteady heat transfer of a vertical helical tube. Various Rayleigh numbers, surface emissivity, coil-to-rod diameter ratios, and pitch-to-rod diameters were examined. A comparison between steady and unsteady behavior is made by analyzing the temperature distribution and changes in the Nusselt number during the cooling process. Correlations to predict *Nu* and *f* were proposed. The thermofluid performance of the twisted tube at various geometries was presented by Ghazanfari et al.^8^. The contribution of Re, pitch length, and twist orientation was examined. These results demonstrate that twisted tubes perform better thermally and hydraulically in helical tube applications, particularly at lower Reynolds numbers. Ghazanfari et al.^9^ studied numerically the effect of nanofluid (Al₂O₃, Cu, CuO, and TiO₂) on enhanced heat transfer of a twisted tube. Due to the increased flow disruption and contact surface area, the results demonstrated that shorter twisted pitch lengths enhanced heat transmission. The ideal pitch length was determined to be 45 mm. Although increasing the volume percentage of nanoparticles increased thermal efficiency, it also increased pressure drop. Compared to other nanoparticles, Cu nanoparticles produced a greater pressure drop but had the best thermal efficiency. Overtaking CuO and TiO₂ nanoparticles, Al₂O₃ nanoparticles demonstrated the least pressure loss while retaining a comparatively good thermal efficiency. Jasim et al.^10^ achieved the thermofluid properties of a shell and twin coiled tube with a hybrid nanofluid. The study covered the effect of hybrid TiO₂-MgI and AG-HEG-based nanofluids. The outcomes are compared to the results of pure water. The findings reveal that the water/MgO-TiO₂ model offers the most exceptional value of thermal performance.

Moghadam et al.^11^ evaluate the impact of phase change material and shell geometry of a helical tube on thermal and exergy performance. Three different shell geometries, including cubic, spherical, and trapezoidal, were examined with different flow rates. According to the findings, the trapezoidal shell reduces the melting time by 23.38 and 10.1%, respectively, in comparison to the cubic and spherical shells. Duan et al.^12^ present a new design of *STHTHX*. The heat and fluid flow parameters, including *Nu*, *f*, and *η*, are used to quantitatively examine the effects of *Re*, corrugation height, and corrugation length on flow and heat transfer performance. The results show that, in comparison to a helically coiled smooth tube heat exchanger, the *Nu*_*i*_ and *Nu*_*sh*_ are up to 2.33 and 2.16 times higher, respectively. Sundar et al.^13^ evaluated MXene nanofluids using water and ionic liquid combinations to determine pumping power, heat transfer, and the second law of thermodynamics in *SSHTHX*. Yu et al.^14^ enhanced the heat transfer efficiency in a shell and corrugated tube. The optimized structure’s friction coefficient dropped by 9.3%, and its Colburn coefficient rose by 5.1% when compared to the original and optimized tube’s results. Lastly, a qualitative comparison of the internal flow field from velocity, pressure, and temperature is made. Yuan et al.^15,16^ presented a novel double shell-passes multi-layer helically tubed design. The impact of coil torsion and coil diameter and pitch on heat exchanger performance as well as varying operating conditions was examined in the 0.0637–0.3183 range and contrasted with traditional multi-layer helically coiled tubes. The findings show that the total heat transfer coefficient is more influenced by the coil pitch and that the impacts of coil pitch changes in the inner and outer helically coiled tubes exhibit distinct patterns^15^. The thermal efficacy and heat transfer rate rose from 5.1% to 12.9%. The shell-side heat transfer coefficient rose from 36.2 to 47.5%, while the total heat transfer coefficient improved from 21.5 to 29.0%^16^. The impact of various spiral coils on the thermofluid behavior in a shell and coil was presented by Mir et al.^17^. Three spiral coils with winding pitches a simple winding were examined. A significant enhancement occurred with decreasing the pitch size. Rajhi et al.^18^ presented the thermofluid characteristics of a shell and two-coil with different outer helical fins and various hybrid nanofluid. The results presented a superior thermal performance of the coil tube with a twisted tape and the SWCNT-MWCNT/Water hybrid nanofluid.

A cmparative investigation was presented experimentally by Abdelghany et al.^19^. The effect of the coil torsion on the shell and coil design thermal performance was investigated at various configurations. The results observed that the thermal characteristics enhanced with torsion decrease. The exergy analysis of a shell and helical tube was examined by Alimoradi et al.^20^ for different geometrical and operational parameters. With a rise in the fluid’s dimensionless inlet temperature differential, the efficiency falls linearly. A correlation was created based on the findings to forecast the effectiveness. Alklaibi et al.^21^ investigate experimentally the effectiveness and heat transfer of the ethylene glycol mixture-based Fe₃O₄-water nanofluid in a shell and helical tube. At 1.0 vol%, the nanofluid resulted in a 21% increase in energy efficiency; however, the benefit becomes less pronounced as the volume concentration increases. The thermophysical characteristics of the nanofluid were correlated. Çolak et al.^22^ used machine learning to forecast the mass flow rate, curvature ratio, tube and coil diameters, Re and De values, and other heat transfer parameters for shell and helically coiled tube heat exchangers. The artificial neural network architectures that were developed calculated the performance evaluation criterion, pressure drop, *Nu*, and inner heat transfer coefficient. Han et al.^23^ investigate heat transfer, exergy loss number, and application in optimization of a shell and helically tube. The effect of the diameter of the tube core, shell, and core tube distance; coiled tube inner diameter and thickness; and mass flow rate on the effectiveness, exergy loss number, and *NTU* was examined. Kirkar et al.^24^ investigated the impact of a helically corrugated tube curvature ratio for a range of 0.0958 to 0.2875 on the thermal–hydraulic performance.

The twisted tubes exhibit a considerable improvement in thermohydraulic performance by 270% compared to the smooth and straight one. The performance of *SSHTHX* with various inclination angles was depicted by Maghrabie et al.^25^. The findings show that the effectiveness of vertical direction efficacy is 26.3% more than its horizontal direction effectiveness. In order to assess *Nu* and *ΔP* as functions of *De* and *θ*, correlations are shown. Miansari et al.^26^ investigate numerically the effects of a novel grooved shell and helical tube on the thermal performance. The study was carried out for the same helical tube pitch with different annulus shell depths. A better thermal performance of up to 20% due to using the grooves of both the inner and outer shell walls compared to the outer grooved shell wall design only.

The higher groove depths significantly increase the thermal performance. Omidi et al.^27^ studied the impact of using various geometrical lobed cross sections, coil pitch, height, and diameter, as well as different fluids (Prandtl number) besides Al₂O₃-water nanofluid, on the performance of helical tubes. A correlation for predicted *Nu* is presented. Darzi et al.^28^ investigated the thermal performance of coiled tubes with various helical corrugated wall parameters. Heat transfer and pressure drop are examined in relation to *Re*, tube diameter, corrugated height, and pitch. The findings indicate that the *Re* raises the *Nu*, although the relative *Nu* falls along with it. With a higher friction factor, heat transmission is improved by higher corrugated height and lower corrugated pitch.

Dizaji et al.^29^ presented the exergy loss analysis of a twisted tube in a twisted shell. Both energy loss and *NTU* increase as a result of tube corrugations. For both the twisted tube and shell design, the energy loss and *NTU* increase by 17–81% and 34–60%, respectively. Salem^30^ examines the thermofluid performance of MWCNT/water nanofluid in the semi-circular tube in a shell. The semi-circular tube spacing ratio, MWCNT volume concentrations, and the operational conditions are the main parameters. The performance characteristics are greatly impacted by the semicircular tube spacing and MWCNT loading. The *η* records 2.46. Compared to the base fluid, the exergy efficiency and thermal performance factor are enhanced by 34.04% and 1.384 times, respectively, at 1.0 weight percent. In previous studies, Abdelmagied^4,31,32^ used a combination of methodologies to examine the properties of twisted helical tubes with different twisted pitch ratios and cross-sectional areas in double and triple tube heat exchangers. The effect of twisted pitch ratio and cross-sectional shape was presented. The results obtained showed that there was a notable improvement in the heat transfer properties due to using the twisted tube. Among the innovative configurations proposed in recent years, the twisted helical tube has attracted significant attention. The proposed compound structure incorporating the tube twisting with helical geometry outperforms straight tubes owing to curvature-induced Dean vortices, which improve mixing and disrupt boundary layers. The study facilitates an experimental analysis of the thermofluid and exergy characteristics of the *STHTHX*. This paper aims to investigate the impact of various twisted helical coil diameters (from 0.095 to 0.165 m), shell-side mass flow rates (from 0.067 to 0.175 kg/s), and twisted helical tube mass flow rates (from 0.033 to 0.181 kg/s), as well as a comparison between *STHTHX* and a conventional *SSHTHX* in counterflow under turbulent conditions, utilizing $\overline{{Nu_{i} }}$, *f*_i,_ *U*_*o*_, *η, λ, η*_*ex*_*,* and* ζ*. The water is employed as a working fluid for both shell and twisted helical coil sides. A comprehensive investigation of these factors is essential to optimize the design and operation of *SSHTHXs*. The study expects to fill the research gap concerning the thermofluid performance of such *STHTHX* designs. Such improvements are expected not only to enhance thermal performance but also to provide practical benefits in refrigeration and air-conditioning applications, where balancing effective heat transfer with acceptable pressure losses remains a key engineering challenge.

## The experimental test rig

**Fig. 1:**
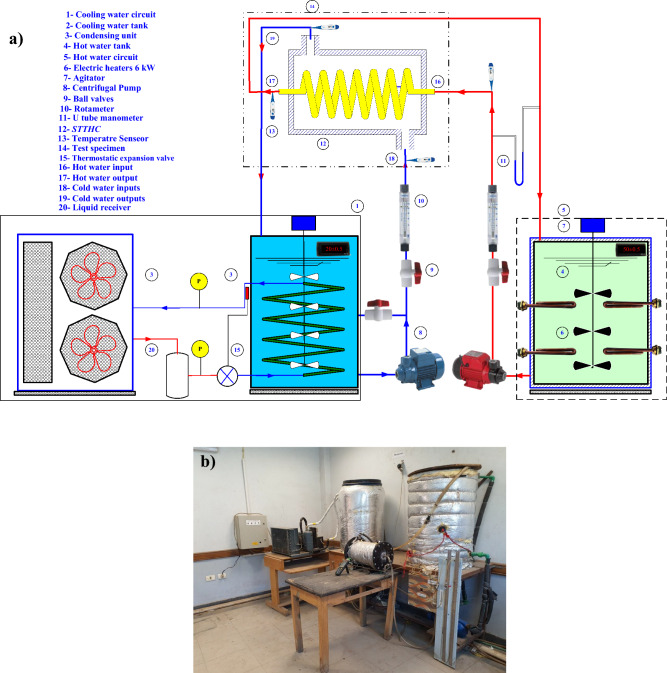
The experimental the test rig. (**a**) Schematic diagram (the schematic drawing in the figure was created by the author using Microsoft Visio 2003, Microsoft Corporation; https://www.microsoft.com/Visio, and (**b**) Photographic picture.

**Fig. 2:**
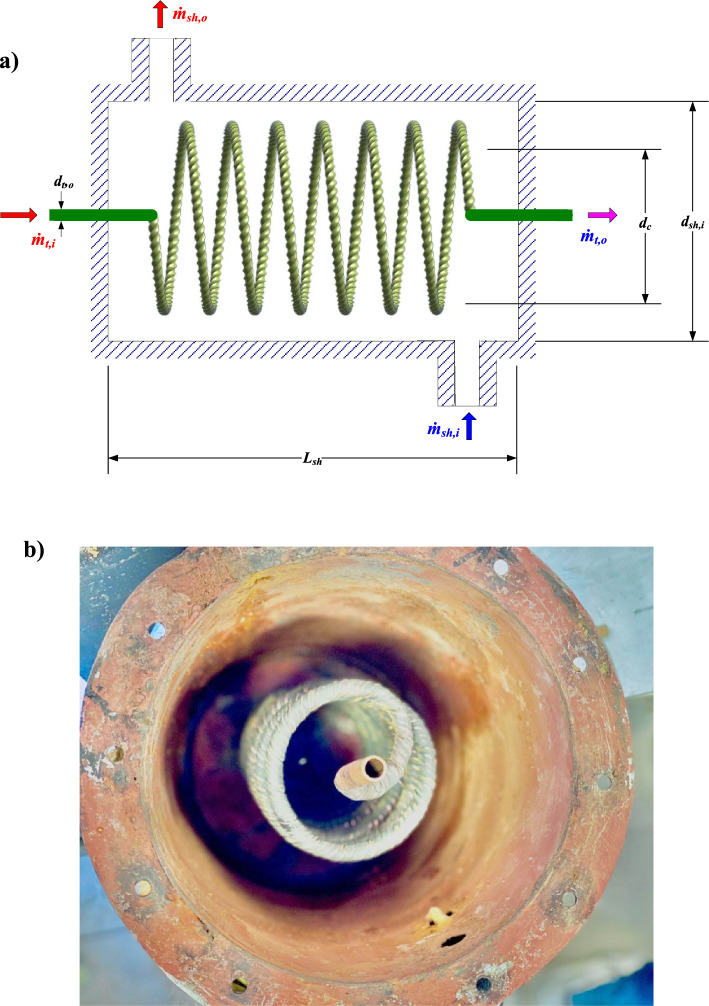
Geometries and dimensions of *STHTHX* profiles (**a**) schematic layout (the schematic drawing in the figure was created by the author using Microsoft Visio 2003, Microsoft Corporation; https://www.microsoft.com/Visio, while the test specimen was created using ANSYS Commercial Version 2019; ANSYS Inc.; https://www.ansys.com), and (**b**) a photograph of the test section.

**Fig. 3:**
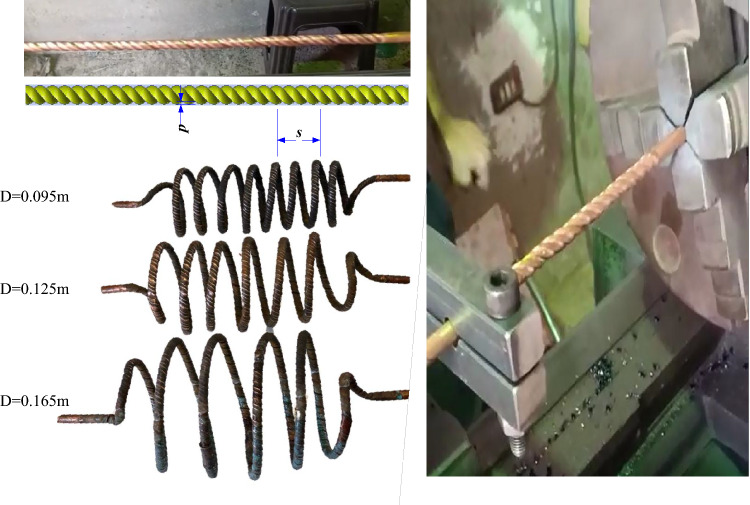
Photograph of forming of *STHTHX* twisted helical tube^4^ (the schematic drawing in the figure was created by the author using ANSYS Commercial Version 2019; ANSYS Inc.; https://www.ansys.com).

The *STHTHX* test rig is shown in Fig. 1(a) and (b). It is consists of a water cooling closed loop, a hot water closed loop, and a *STHTHX* loop. The components of the closed-circuit water cooling system include an adjustable temperature control, a 0.2m^3^ insulated tank, and a 1.5 kW cooling circuit. An insulated 0.2m^3^ tank, an adjustable temperature control mechanism, and electric heaters with a combined 6 kW capacity make up the closed hot water circuit components. A twisted helical copper tube coil (ρ of 8978 kg/m^3^, C of 381 J/kg.^o^C, and k of 387.6 W/m. K) and a stainless steel cylindrical tube with *D*_*sh,i*_ of 0.2268 m diameter and 0.4 m length make up the shell side of the *STHTHX* loop. As shown In Fig. 2, where *d*_*c*_ and *d*_*t,o*_ stand for *STHTHX* twisted helical coil diameter and tube outer diameter, respectively, three twisted helical coils are formed on a lathe^4^ (Fig. 3) and are helically wrapped at a constant diameter of 0.095, 0.125, and 0.165 m with a conventional smooth helical coil tube of 0.125 as a particular reference. The working fluid is water in both the twisted helical tube and shell sides in counterflow arrangements. The water in the twisted helical tube side, is pumped to enter the test section from the left to the right, while the water in the shell side is pumped to enter from the bottom to the top of the shell. The geometrical dimensions and key design factors are summarized in Table 1. A 1 horsepower centrifugal pump supplied the hot water to the *STHTHX* through a twisted helical tube, and a ball valve and rotameter (scale resolution of 0.5L/min) to adjust the flow rate, which ranged from 0.016 to 0.3 kg/s. The temperature of the hot water is supplied at 50 °C + /- 0.5 °C. Additionally, a centrifugal pump with a 1 horsepower capacity pumps the cold water into the *STHTHX* shell side at a temperature of 20 °C + /− 0.5 °C. A rotameter (scale resolution of 0.5L/min) and a ball valve were used to adjust the flow rates, which ranged from 0.016 to 0.3 kg/s. A *STHTHX*’s shell has thermal insulation. Four pre-calibrated K-type thermocouples were employed to monitor the temperatures of the *STHTHX* intake and exit fluids. In addition, twelve thermocouples were supported on the outer surface tube wall of the helical coil to measure the surface temperatures. All thermocouples were connected to a digital thermometer indicator with a resolution of 0.1 °C to display the thermocouples outputs. A U-tube manometer (with an accuracy of ± 1 mmHg) was used to measure the pressure decrease over the *STHTHX*. The system was allowed to monitor the stability of the measurement parameters (temperatures and pressure drop) for around 40 min in order to reach the steady-state condition.

**Table 1:** Shell and twisted helically tube geometry parameters and operating condition.

Geometry characteristics	Helical tube	Shell side
Outer diameter, *d*_*o*_ (mm)	10.7	250
Inner diameter, *d*_*i*_ (mm)	9.28	228.6
Curvature ratios, *δ* = *d*_*t,i*_*/d*_*c*_	0.098, 0.074, 0.056	-
coil diameter, *d*_*c*_ (mm)	95, 125, 165	-
Twisted height, *p*, (mm)	1	-
Twisted pitch, *s*, (mm)	36	-
Length, *L*(mm)	3000	400
Number of turns, *N*	9, 6, 5	-
Helical coil material	Copper	Steel
Fluid feeding velocity, m.s^-1^	0.49:2.7	0.0016:0.047
Fluid feeding temperatures, ^o^C	50 ± 0.5 °C	20 ± 0.5 °C

## Error analysis

When incorporating the mistake in the findings, the experimental error’s uncertainty should be taken into account. Error analysis was conducted using a differential approximation approach^33^.

For the independent variables (*s*_*i*_, *s*_*2*_, *s*_*3*_,…, *s*_*n*_), considering the uncertainty in *ω*_*1*_, *ω*_*2*_, …, *ω*_*n*_, and *ω*_*R*_,

1 $$\omega_{R} = \sqrt {\left( {\frac{\partial R}{{\partial s_{1} }}\omega_{1} } \right)^{2} + \left( {\frac{\partial R}{{\partial s_{2} }}\omega_{2} } \right)^{2} + \,\,...\,\, + \left( {\frac{\partial R}{{\partial s_{n} }}\omega_{n} } \right)^{2} }$$

Table 2 presents the findings of the uncertainty measurements.

**Table 2:** The uncertainties values of measuring devices and study parameters.

Instruments	Range	Accuracy (%)	Uncertainty (%)
Rotameter, kg/s	0.03–0.181	± 0.5	-
K-type thermocouple, ^o^C	-200–1200	± 0.1	-
U tube manometer, mm Hg	0.5–500	± 1	-
*Re*_*i*_	44000–6100	-	± 2.87
*f*_*i*_	-	-	± 3.1
$\overline{{h_{i} }}$_,_ W.m^-2^.^o^C^-1^	-	-	± 3.27
$\overline{{Nu_{i} }}$	-	-	± 3.29
*η*	-	-	± 2.1
*η*_*ex*_	-	-	± 2.7

## Data reduction

The thermofluid characteristics of the *STHTHX* can be obtained by defining the heat supplied from the hot fluid and the heat absorbed by the cold fluid can be expressed as:

2 $$\mathop {Q_{t} }\limits^{.} = \mathop {m_{t} }\limits^{ \cdot } C_{t} \left( {T_{t,\,i} - T_{t,\,o} } \right)$$

3 $$\mathop {Q_{sh} }\limits^{.} = \mathop {m_{sh} }\limits^{ \cdot } C_{sh} \left( {T_{sh,\,o} - T_{sh,\,i} } \right)$$

The following equation can be expressed the overall heat transmitted coefficient:

4 $$\,U_{o} = \frac{{\mathop {Q_{Avg.} }\limits^{.} }}{{\pi d_{i,o} L\,\delta T_{LMTD} }}$$

where the average rate of heat transfer can be calculated as.

*Q*_*Avg.*_ = *0.5*(*Q*_*t*_ + *Q*_*sh*_) (5)

6 $$\delta T_{LMTD} = \frac{{\left( {T_{t,i} - T_{sh,o} } \right) - \left( {T_{t,o} - T_{sh,i} } \right)}}{{\ln \left[ {{{\left( {T_{t,i} - T_{sh,o} } \right)} \mathord{\left/ {\vphantom {{\left( {T_{t,i} - T_{sh,o} } \right)} {\left( {T_{t,o} - T_{sh,i} } \right)}}} \right. \kern-0pt} {\left( {T_{t,o} - T_{sh,i} } \right)}}} \right]}}$$

The heating load of the *STHTHX*, Q_avg_, was used to calculate the average heat transfer coefficient,$\overline{{h_{i} }}$, for the tube side in the *STHTHX*, and then the average Nusselt number, $\overline{{Nu_{i} }}$, as follows:

7 $$\mathop Q\limits^{.}_{avg} = \overline{{h_{i} }} A_{t,i} \left( {T_{t,b} - \overline{T}_{t,s} } \right)$$

where

8 $$T_{t,b} = \frac{{\left( {T_{t,i} + T_{t,o} } \right)}}{2}$$

9 $$T_{t,s} = \frac{{\sum {T_{t,s} } }}{12}$$

10 $$\overline{{Nu_{i} }} = \frac{{\overline{h}_{i} d_{h,ti} }}{{k_{i} }}$$

By calculating $\overline{{h_{i} }}$, the annulus side convective heat transfer coefficient,$\overline{{h_{sh} }}$_*,*_ can be determined from the overall heat transfer coefficient relationship as^34^:

11 $$\frac{1}{{\mathop U\nolimits_{o} }} = \frac{{\mathop A\nolimits_{o} }}{{\mathop A\nolimits_{i} \overline{{\mathop h\nolimits_{i} }} }} + \frac{{\mathop A\nolimits_{o} \ln \left( {{{\mathop d\nolimits_{o} } \mathord{\left/ {\vphantom {{\mathop d\nolimits_{o} } {di}}} \right. \kern-0pt} {di}}} \right)}}{2\pi kL} + \frac{1}{{\overline{{\mathop h\nolimits_{sh} }} }}$$

12 $$Nu_{sh} = \frac{{\overline{h}_{sh} d_{h,sh} }}{{k_{sh} }}$$

where the hydraulic diameter* d*_*h,sh*_ in the* STHTHX* shell side and can be defined as according to Shah and Sekulic^35^, as:

13 $$d_{h,sh} = \frac{{4\left( {V_{sh,i} - V_{t,o} } \right)A_{{c.{\mathrm{s}} }} }}{{\,A_{t,o} }} = \frac{{4\left[ {{\raise0.7ex\hbox{$\pi $} \!\mathord{\left/ {\vphantom {\pi 4}}\right.\kern-0pt} \!\lower0.7ex\hbox{$4$}}\left( {d_{sh,i}^{2} L_{sh} - d_{{_{t,\,o} }}^{2} L_{t} } \right)} \right]}}{{\pi \left( {d_{sh,i} L_{sh} + d_{t,\,o} L_{t} } \right)}} = \frac{{d_{sh,i}^{2} L_{sh} - d_{t,\,o}^{2} L_{t} }}{{d_{sh,i} L_{sh} + d_{t,\,o} L_{t} }}$$

The Reynolds number for the twisted helical tube hot water (*Re*_*i*_) and for the shell-side cold water (*Re*_*sh*_) can be calculated as follows:

14 $$Re_{i} = \frac{{4\mathop{m_{i} }\limits^{.} \,}}{{\pi d_{t,i\,} \mu_{i} }}$$

15 $$Re_{sh} = \frac{{4\mathop{m_{sh} }\limits^{.} \,}}{{\pi d_{h,sh\,} \mu_{sh} }}$$

The friction factor, *f*_*i*_*,* of the *STHTHX* is expressed as:

16 $$f_{i} = \frac{{2\delta P_{i} }}{L}.\frac{{d_{h,t} }}{{\rho v_{i}^{2} }}$$

The thermal performance index (*η*) of the *STHTHX* can be estimated according to Webb^36^ as:

17 $$\eta = {{\frac{{Nu_{Twistet} }}{{Nu_{Smooth} }}} \mathord{\left/ {\vphantom {{\frac{{Nu_{Twistet} }}{{Nu_{Smooth} }}} {\left( {\frac{{f_{Twistet} }}{{f_{Smooth} }}} \right)^{1/3} }}} \right. \kern-0pt} {\left( {\frac{{f_{Twistet} }}{{f_{Smooth} }}} \right)^{1/3} }}$$

The exergy balance equation for a *STHTHX* can be expressed by Alimoradi^18^ as:

18 $$\mathop {E_{in} }\limits^{.} = \mathop {E_{out} }\limits^{.} + \mathop {E_{loss} }\limits^{.}$$

19 $$\mathop {E_{in} }\limits^{.} = m_{i} \left[ {C\left( {T_{t,i} - T_{0} } \right) - CT_{0} Ln\left( {\frac{{T_{t,i} }}{{T_{0} }}} \right) + \frac{{v_{t}^{2} }}{2}} \right] + m_{sh} \left[ {C\left( {T_{sh,i} - T_{0} } \right) - CT_{0} Ln\left( {\frac{{T_{sh,i} }}{{T_{0} }}} \right) + \frac{{v_{sh}^{2} }}{2}} \right]$$

20 $$\mathop {E_{out} }\limits^{.} = m_{i} \left[ {C\left( {T_{t,o} - T_{0} } \right) - CT_{0} Ln\left( {\frac{{T_{t,o} }}{{T_{0} }}} \right) + \frac{{v_{t}^{2} }}{2}} \right] + m_{sh} \left[ {C\left( {T_{sh,o} - T_{0} } \right) - CT_{0} Ln\left( {\frac{{T_{sh,o} }}{{T_{0} }}} \right) + \frac{{v_{sh}^{2} }}{2}} \right]$$

Hence, the *STHTHX* exergy efficiency, *η*_Ex._, can be written as:

21 $$\eta_{Ex.} = \frac{{\mathop {E_{out} }\limits^{.} }}{{\mathop {E_{in} }\limits^{.} }}$$

The *STHTHX* exergy destruction rate can be formulated as:

22 $$\lambda = \mathop {E_{in} }\limits^{.} - \mathop {E_{out} }\limits^{.}$$

The exergy dimensionless ratio can be calculated as follows^37^:

23 $$\zeta = \frac{\lambda }{{\left( {\mathop m\limits^{.} C} \right)_{\min } T_{e} }}$$

The *STHTHX* critical Reynolds number, *Re*_*cr,i*_, for tube side is expressed as^38^:

24 $${\mathrm{Re}}_{cr,i} = 30000\,\delta_{i}^{0.53} ,\,\,\,\,\,\,\,\,\,\delta_{i}^{ - 1} \le \,24$$

## Results and discussion

### Validation of the experimental work

**Fig. 4:**
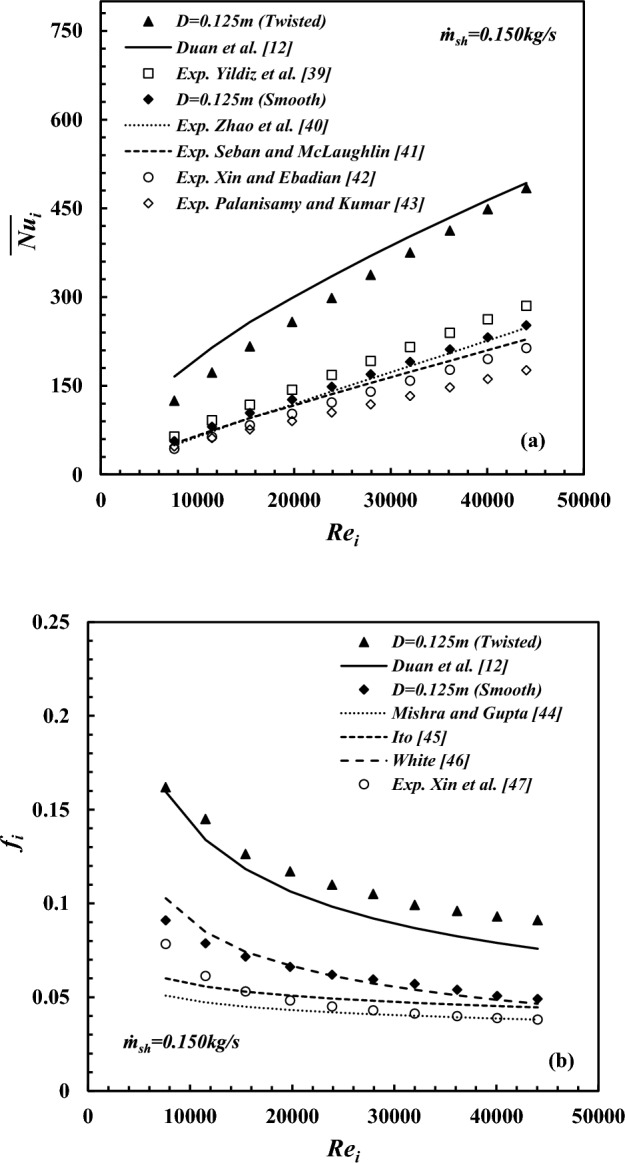
Validation of the present results of *STHTHX* with open literature (**a**) *Nu*_*i*_ against *Re*_*i*_ and (**b**) *f*_*i*_ against *Re*_*i*_.

The experimental study was verified by contrasting the findings of $\overline{{Nu_{i} }}$ for water passing through the* STHTHX* with $\overline{{Nu_{i} }}$ those published in the literature. From Fig. 4a, b and Table 3, the present study was compared for both twisted and smooth helical coils of the *STHTHX* with the data of the open literature. It is obvious that there is a good agreement between the results of $\overline{{Nu_{i} }}$ for the *STHTHX* twisted helical tube side and the correlations of Duan et al.^12^ and Yildiz et al.^39^, with an average deviation of 11 and 76.9%, while the *SSHTHX* were assessed with Zhao et al.^40^, Seban and McLaughlin^41^, Xin and Ebadian^42^, and Palanisamy and Kumar^43^ with an average deviation of 5.2, 9.4, 10.4, and 12.4%, respectively.

**Table 3:** The mean, maximum and minimum Error values of the comparison of the experimental results with published work.

Smooth tube											Twisted tube					
Nu						*f*					Nu				*f*	
Present study		^40^	^41^	^42^	^43^	Present	^44^	^45^	^46^	^47^	Present		^12^	^39^	Present	^12^
Re											Re					
7616	56.3	49.5	52.5	43.3	47.3	0.091	0.0509	0.0600	0.1027	0.072	7616	124.6	165.8	63.9	0.162	0.1595
11508	80.7	72.8	74.7	63.6	61.5	0.079	0.0472	0.0557	0.0848	0.057	11508	172.1	214.7	91.5	0.145	0.1339
15431	103.9	95.7	95.7	83.2	75.7	0.072	0.0449	0.0529	0.0741	0.051	15431	216.5	257.9	117.8	0.126	0.1183
19801	126.3	118.1	116.0	102.5	90.0	0.066	0.0433	0.0509	0.0668	0.046	19801	258.0	298.4	143.2	0.117	0.1064
23911	148.4	140.4	135.9	121.7	104.5	0.062	0.0419	0.0493	0.0614	0.043	23911	298.3	335.3	168.2	0.11	0.0982
27940	169.4	161.9	154.8	140.0	118.3	0.059	0.0409	0.0481	0.0573	0.041	27940	337.4	369.9	191.9	0.105	0.0919
32006	190.3	183.4	173.5	158.5	132.4	0.057	0.0401	0.0470	0.0539	0.04	32006	375.3	402.7	215.6	0.099	0.0868
36147	211.3	205.2	192.2	177.1	147.1	0.054	0.0393	0.0461	0.0510	0.039	36147	412.2	434.2	239.2	0.096	0.0824
40070	231.6	226.5	210.3	195.2	161.4	0.051	0.0387	0.0453	0.0486	0.038	40070	448.6	464.0	262.2	0.093	0.0789
44043	252.1	248.0	228.4	213.5	176.1	0.049	0.0381	0.0445	0.0465	0.037	44043	484.1	492.9	285.1	0.091	0.0758
Max. Err	-	13.9	10.3	30.0	43.6	-	78.7	51.5	5.8	44.1	-	-	33.1	94.9	-	20.0
Min. Err	-	1.6	7.4	18.1	19.0	-	28.6	10.0	-13	25.6	-	-	1.8	69.8	-	1.5
Avg. Err	-	5.2	9.4	10.4	12.4	-	45.6	23.9	1.5	39.7	-	-	11.0	76.9	-	13.3

On the other side, the results of *f*_*i*_ were also validated for both the twisted and smooth helical coils of the *STHTHX* with the data of the open literature (Fig. 4b). It is obvious that there is a good agreement between the results of *f*_*i*_ for the *STHTHX* twisted helical tube side and the correlation of Duan et al.^12^ with an average deviation of 13.3%, while the *STHTHX* were assessed with Mishra and Gupta^44^, Ito^45^, White^46^ and Xin et al.^47^ with an average deviation of 45.6, 23.9, 1.5 and 39.7%, respectively.

### Impact of shell-side mass flow rate

**Fig. 5:**
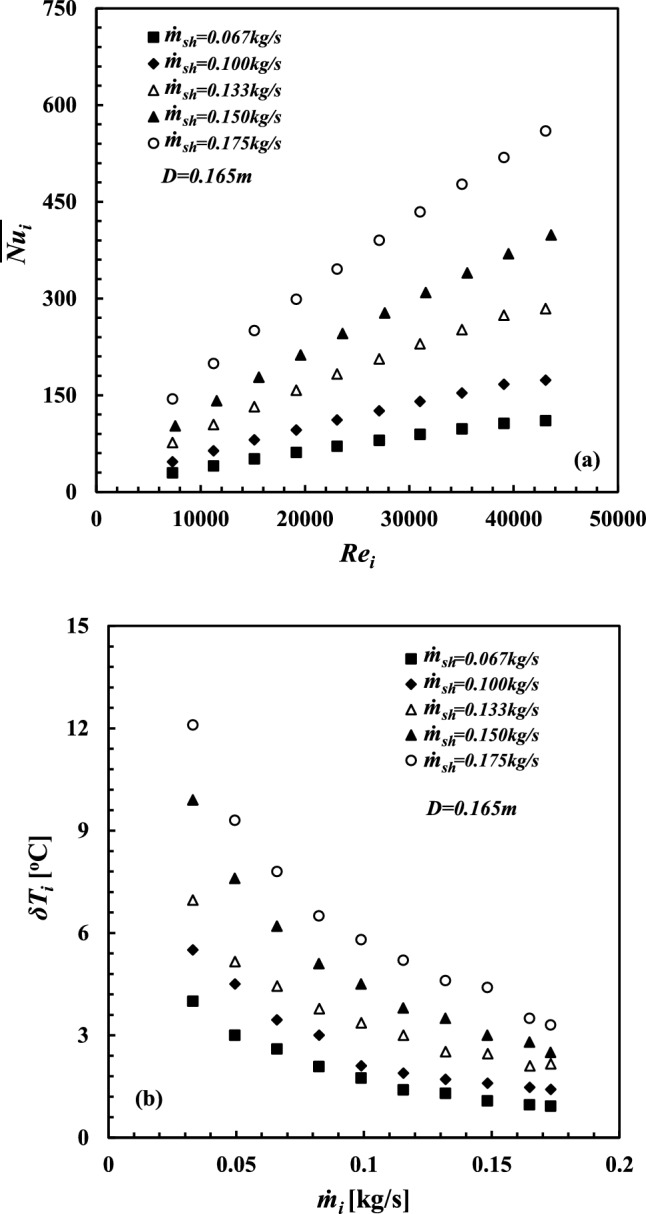
Variation between *TTTHC* and *TDTHC* on (**a**) $\overline{{Nu_{i} }}$ against *Re*_*i*_ and (**b**) *δT*_*i*_ against *ṁ*_*i*_.

To explore the impact of shell-side mass flow rate on the *STHTHX* thermal characteristics, Fig. 5a shows the relation between the $\overline{{Nu_{i} }}$ versus *Re*_*i*_ for a range of 7300 ≤ *Re*_*i*_ ≤ 43,600 at various shell-side water mass flow rates of 0.067 ≤ $\mathop {m_{sh} }\limits^{.}$ ≤ 0.175 kg/s as well as a test specimen of *D* = 0.165 m. Figure 5a illustrates that the-shell side mass flow rate has a noticeable effect on $\overline{{Nu_{i} }}$ of the *STHTHX*. For the same value of *Re*_*i*_ of 31000, the shell-side mass flow rate of 0.175 kg/s presents an increase in $\overline{{Nu_{i} }}$ by 3.87 times higher than that of 0.067 kg/s. This can be revealed by the increase in the average heat transfer rate due to the increase in in the shell-side water mass flow rate as the temperature difference f increases for both hot and cold fluids. This means increasing the hot water temperature differences, convective heat transfer coefficient, and heat transfer rate and consequently increasing $\overline{{Nu_{i} }}$.

Figure 5b depicts the impact of various shell-side water mass flow rates on the *STHTHX* hot water temperature differences. It can be noticed that the hot water in the twisted helical tube decreases with the increase of water mass flow rate. As *ṁ*_*sh*_ grows, the *STHTHX* heat transmitted rate enhanced and increasing the hot water temperature disparities for the same *ṁ*_*i*_. For the same *ṁ*_*i*_ of 0.13 kg/s, the shell-side mass flow rate of 0.175 kg/s is 2.5 times higher than that of 0.067 kg/s.

### Impact of the *SSHTHX *and the* STHTHX*

**Fig. 6:**
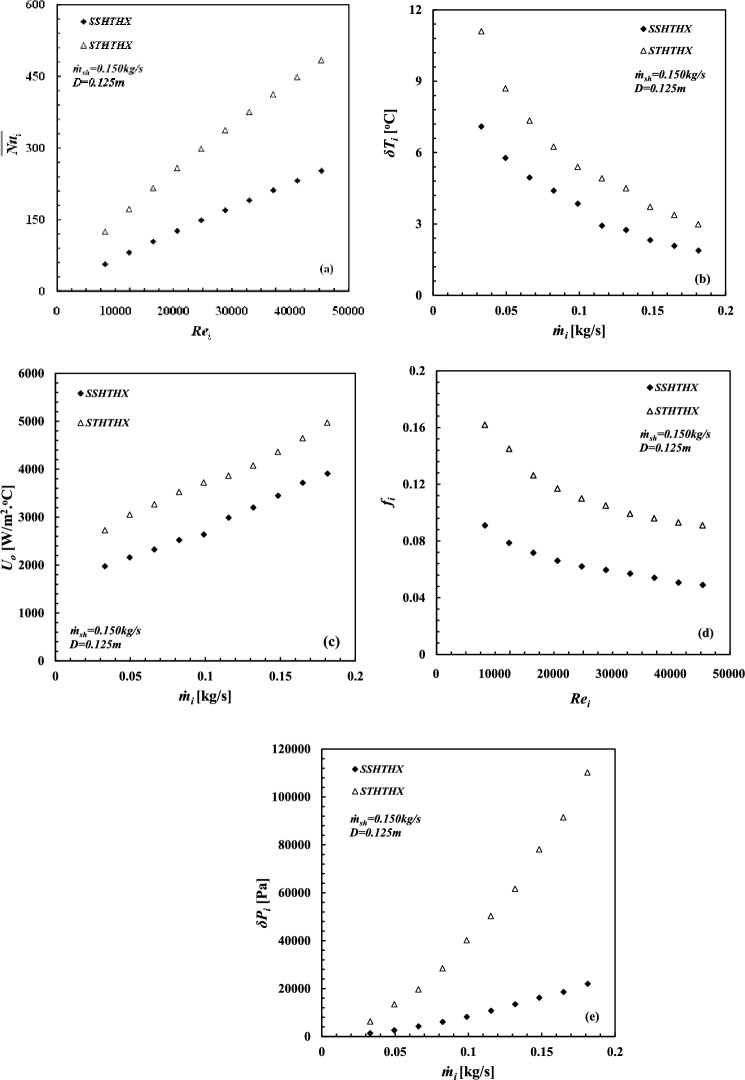
Variation between *STHTHX* and *SSHTHX* on (**a**) $\overline{{Nu_{i} }}$ vs. *Re*_*i*_, (**b**) *δT*_*i*_ vs. *ṁ*_*i*_, (**c**) *U*_*o*_, vs. *ṁ*_*i*_, (**d**) *f*_*i,*_ vs *Re*_*i*_, and (**e**) *δP*_*i*_, vs. *ṁ*_*i*_.

To achieve the impact of the shell and smooth helical tube heat exchanger, *SSHTHX,* and the shell and twisted helical tube heat exchanger,* STHTHX,* thermal characteristics, the experimental results were carried out for both smooth and twisted helical coils at the same test specimen dimensions of *D* = 0.125 m and at the same twisted coil length of 3 m. Figure 6a illustrates the variation of $\overline{{Nu_{i} }}$ versus *Re*_*i*_ between both *SSHTHX* and *STHTHX* designs for a range of 6000 ≤ *Re*_*i*_ ≤ 43,600 and at shell-side mass flow rate of 0.15kgs. From Fig. 6a it can be indicated that the $\overline{{Nu_{i} }}$ of both *SSHTHX* and *STHTHX* significantly increases with increasing of *Re*_*i*_ and the *STHTHX* presents superior thermal hydraulic performance. When *Re*_*i*_ rises from 6.1 × 10^3^ to 4.4 × 10^4^, the total increments in $\overline{{Nu_{i} }}$ for both *STHTHX* and *SSHTHX* with *D* = 0.125 m are 288.7, and 347.4%, respectively. Comparatively, the $\overline{{Nu_{i} }}$ of *STHTHX* optimally outperforms *SSHTHX*. Furthermore, the average growth in $\overline{{Nu_{i} }}$ for *STHTHX* to *SSHTHX* is 102.5%. While the flow in the helical tube produces a secondary flow due to the generation of the centrifugal force, which moves the fluid particles toward the outer wall of the tube and raises the thermal characteristics, the twisted helical tube adds an additional axial flow, which producing a larger vortex region on the tube wall along the axis of the flow. This combination of the secondary flow and the axial flow leads to a rise in the fluid temperature difference (Fig. 6b).

Figure 6c illustrates the relation between the overall heat transfer coefficient, *U*_*o*_, versus *ṁ*_*i*_ for a range of 0.033 ≤ *ṁ*_*i*_ ≤ 0.181 and at shell-side mass flow rate of 0.15kgs. From Fig. 6c it can be indicated that *U*_*o*_ significantly increases with increasing *ṁ*_*i*_. When *ṁ*_*i*_ increases from 0.033 to 0.181 kg/s, the total increments in the* U*_*o*_ for both *STHTHX* and *SSHTHX* with *D* = 0.125 m are 82.4, and 98.1%, respectively. Comparatively, the *U*_*o*_ of *STHTHX* presents an optimal performance compared to that of *SSHTHX*. Moreover, the *STHTHX* present an average grow in* U*_*o*_ by 33.7% compared to *SSHTHX*. This can be attributed to the twisted helical tube structure that augments heat transfer characteristics due to producing a strong secondary flow, which results in increasing the vortex growth close to the tube wall. This leads to enhancing the fluid log mean temperature difference and heat transfer rates (Eq. 4) and consequently increasing *U*_*o*_.

Figure 6d illustrates the relation between the twisted helical tube friction factor, *f*_*i,*_ versus *Re*_*i*_ for a range of 7300 ≤ *Re*_*i*_ ≤ 43,600 and at shell-side mass flow rate of 0.15kgs. From Fig. 6d it can be indicated that *f*_*i*_ significantly decreases with increasing *Re*_*i*_ and decreasing the twisted coil diameter, *D*. When *Re*_*i*_ rises from 6.1 × 10^3^ to 4.4 × 10^4^, the total decrements in the *f*_*i*_ for both *STHTHX* and *SSHTHX* with *D* = 0.125 m are 42.1%, and 45.4, respectively. Comparatively to the *SSHTHX* the *f*_*i*_ of *STHTHX* was grown*.* Furthermore, the average growth in* f*_*i*_ for *STHTHX* compared to *SSHTHX* is 84.1%. This increase can be referred to the structure of the twisted helical tubes, which produce a strong secondary flow due to *δ* and a strong vortex region on the twisted tube side of *STHTHX* as aresults of the generation of the axial flow along the main flow axial due to tube twist. This rise in the fluid turbulence through the twisted helical tube leads to growth in the pressure drop difference (Fig. 6e).

### Impact of twisted helical tube diameter

**Fig. 7:**
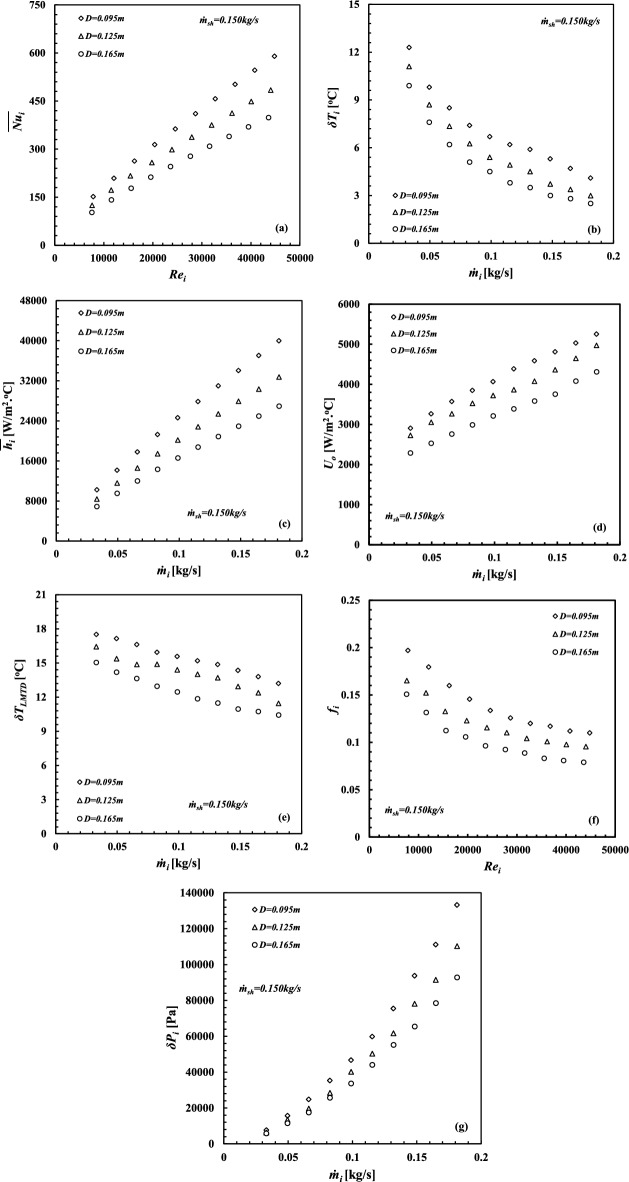
Variation twisted helical coil diameter of *SSHTHX* on (**a**) $\overline{{Nu_{i} }}$ vs. *Re*_*i,*_ (**b**) $\overline{{h_{i} }}$ vs. *Re*_*i*_, (**c**) *δT*_*i*_, vs. *ṁ*_*i*_, (**d**), (*U*_*o*_, vs. *ṁ*_*i,*_ (**e**) *δT*_*LMTD*_, vs. *ṁ*_*i*_, (**f**) *f*_*i*_, vs *Re*_*i*_, and (**g**) *δP*_*i*_, vs. *ṁ*_*i*_.

The consider the impact of twisted helical tube diameter on the *STHTHX* thermal characteristics, three twisted helical coil diameters of 0.095 m, 0.125, and 0.165 m are examined at the same twisted coil length of 3 m. Figure 7a illustrates the relation between the $\overline{{Nu_{i} }}$ versus *Re*_*i*_ for a range of 7300 ≤ *Re*_*i*_ ≤ 43,600 while maintaining a constant shell side mass flow rate of 0.15kgs. From Fig. 7a it can be indicated that $\overline{{Nu_{i} }}$ significantly increases with increasing *Re*_*i*_ and decreasing the twisted coil diameter, *D*. When *Re*_*i*_ rises from 7.57 × 10^3^ to 4.4 × 10^4^, the total increments in the *STHTHX* $\overline{{Nu_{i} }}$ with *D* = 0.095 m, 0.125 m, and 0.165 m are 289.9, 288.7, and 289.2%, respectively. Furthermore, when the coil diameter decreases from *D* of 0.165 to 0.0125 m and *D* = 0.165 m, the average growth in $\overline{{Nu_{i} }}$ are 21.5 and 47.9% respectively. While the coil diameter decreases from 0.165 m to 0.095 m the twisted helical tube curvature ratio rises from 0.056 to 0.0976. This increase in curvature ratio increases the forming of the flow due to the generation of the centrifugal force. This secondary flow moves the fluid particles toward the outer wall of the twisted helical tube, and this leads to enhancing the fluid temperature difference (Fig. 7b) and enhancing the convective heat transfer coefficient (Fig. 7c) and consequently increasing $\overline{{Nu_{i} }}$. On the other hand, the compound structure of the twisted helical tube also exhibits an extra intensity of the secondary flow on the tube side and produces a larger region of fluid vortexes on both the twisted tube sides.

Figure 7d illustrates the relation between the overall heat transfer coefficient, *U*_*o*_, versus *ṁ*_*i*_ for a range of 0.033 ≤ *ṁ*_*i*_ ≤ 0.181 and at shell-side mass flow rate of 0.15kgs. From Fig. 7d it can be indicated that *U*_*o*_ significantly increases with increasing *ṁ*_*i*_ and decreasing the twisted coil diameter, *D*. When *ṁ*_*i*_ rises from 0.033 to 0.181, the total increments in the *STHTHX U*_*o*_ for *D* = 0.095, 0.125, and 0.165 m are 80.74, 82.36, and 88.55%, respectively. Furthermore, when the coil diameter decreases from *D* of 0.165 to 0.0125 m and *D* = 0.165 m, the average growth in *U*_*o*_ is 16.6 and 27.2% respectively. While the twisted coil diameter decreases from 0.165 to 0.095 m the twisted helical tube curvature ratio rises from 0.056 to 0.0976. This increase in curvature ratio beside the twisted tube structure presents a strong secondary flow effect within the twisted helical tube and results in vortex appearance close to the tube wall. This leads to enhancing the fluid log mean temperature difference (Fig. 7e) and enhancing the heat transfer rates and consequently increases* U*_*o*_.

Figure 7f illustrates the relation between the twisted helical tube friction factor, *f*_*i,*_ versus *Re*_*i*_ for a range of 7300 ≤ *Re*_*i*_ ≤ 43600 and at shell-side mass flow rate of 0.15kgs. From Fig. 7f it can be indicated that *f*_*i*_ significantly increases with increasing *Re*_*i*_ and decreasing the twisted coil diameter, *D*. When *Re*_*i*_ rises from 7.57 × 10^3^ to 4.4 × 10^4^, the total decrements in the *STHTHX f*_*i*_ for *D* = 0.095, 0.125, and 0.165 m are 42.4, 42.1, and 47.6%, respectively. Furthermore, when the coil diameter decreases from *D* of 0.165 to 0.0125 m and *D* = 0.165 m, the average growth in* f*_*i*_ is 14.7 and 38.77% respectively. While the coil diameter decreases from 0.165 to 0.095 m the twisted helical tube curvature ratio rises from 0.056 to 0.0976. This increase in curvature ratio beside the structure of the twisted helical tubes produces a strong secondary flow due to δ and a strong vortex region on the twisted tube side by forming the axial flow due to tube corrugation. This raises the fluid turbulence through the twisted helical tube and leads to growth in the pressure drop difference (Fig. 7g).

The results of this study are contrasted with those of related studies on *SSHTHXs* in Table 4. Several variables, including flow rate, Reynolds number, heat transfer rate, and heat transfer coefficient, are shown in Table 4. As is well recognized, the specified parameters are crucial for assessing heat exchangers. A thorough and comprehensive comparison of this work’s results with those of comparable studies reveals a strong degree of agreement between them. It is preferable to clarify that the test circumstances and geometrical characteristics of the specified *SHCTHEXs* differ. It is challenging to perform a sensitive comparison of the provided works because of this phenomena. However, overall, it can be said that the results of this study are consistent with the relevant literature.

**Table 4:** A general comparison between the current study and related works.

Ref	Application	Flow rate	*Re*_*i*_	*h*_*i*_ (W/m^2^·K)	*U*_*o*_ (W/m^2^·K)	*Nu*_*i*_
Jamshidi et al.^48^	Exp. And Num	1–4 L/min	2000–10000	2671 (optimum)	625–1100	40–80
Panahi and Zamzamian^49^	Num	1–5 L/min	4000–18000	–	400–1700	–
Elshazly et al.^50^	Exp. And Num	1.7–11.15 L/min	5700–55000	2000–2700	500–2000	20–270
Bahrehmand and Abbassi^51^	Exp. And Num	0.03–0.113 kg/s	10,000–35000	5000–25000	1000–1550	40–140
Barzegari et al.^52^	Exp	2–3.5 L/min	10,800–21182	2150–3000	–	16–20
Salem et al.^53^	Exp. And Num	1.7–11 L/min	10,000–60000	–	200–1500	50–240
Tuncer et al.^54^	Exp. And Num	1.5–3.5 L/min	6600–16000	5700–13400	1600–3150	53–125
Güngor et al.^55^	Exp. And Num	2–6 L/min	10600–14200	5200–6500	–	–
Ghaderi et al.^56^	Exp	3–6 L/min	9000–26000	5000–22500	–	–
Khanlari et al.^57^	Exp. And Num	1.5–3.5L/min	6655–20880	3400–12500	–	–
This study	Exp	2–11 L/min	7600–44000	4031–40000	1973–5250	56–589

### Thermal performance index and exergy analysis

**Fig. 8:**
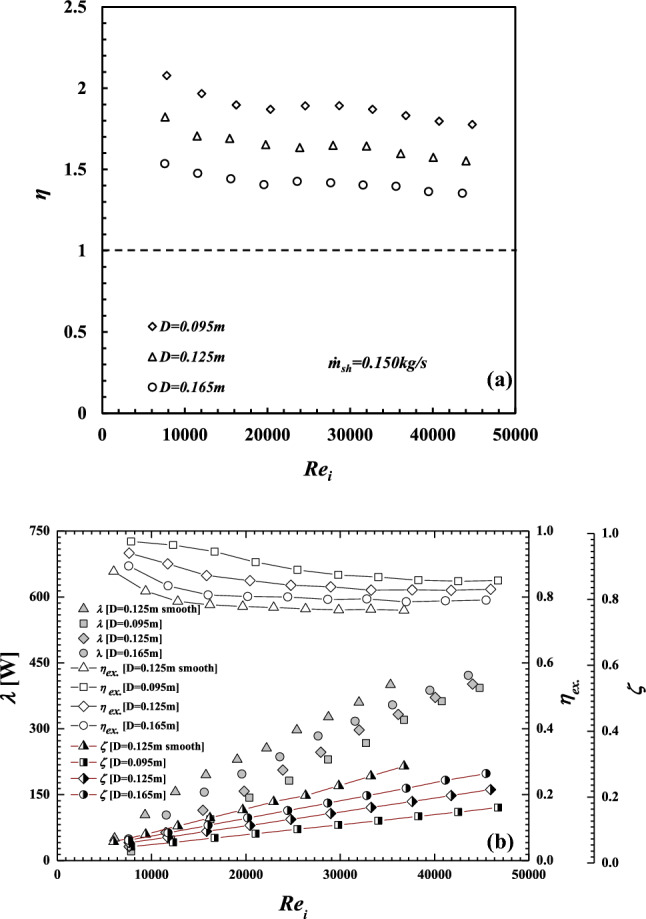
variation of coil diameter of *SSHTHX* and *STHTHX* on (**a**) *η* against *Re*_*i*_ and (**b**) *λ*, *η*_*ex.*_, and* ζ* against *Re*_*i*_.

The thermal performance index, *η*, of the *STHTHX* is a parameter that is used to consider the improvement of the *STHTHX* performance under various curvature ratios. At the same *ṁ*, the *η* can be stated to be the ratio between the Stanton number augmentation ratios to the ratio of *f *^36^. Figure 8a is shows the variation of *η* against *Re*_*i*_ for various twisted tube helical coil diameters. It can be declared from the figure that the *η* is above unity for all twisted helical coil diameters, and the highest *η* is acquired at D = 0.095. Furthermore, as coil diameter decreases, the *η* also increases, implying that the thermal characteristics advantage grows more pronounced compared to the increment in the fluid flow resistance. The maximum values of the *η* record are 2.07 at 1.82 and 1.54 at *D* = 0.095, 0.125, and 0.165 m, respectively.

The second law of thermodynamics may be used to quantify the behavior of the* STHTHX*. One interesting aspect of this study is the *STHTHX* exergy analysis, which is a crucial economic assessment metric for system-wide analysis. Via taking the *STHTHX* system into account as a smaller subset. In these systems, the exergo-economic features of any subset of equipment are taken into consideration when evaluating the system’s exergo-economic characteristics. Figure 8b depicts the variation of exergy performance parameters, including exergy destruction rate, λ, exergy efficiency, *η*_*ex.*_*,* and exergy dimensionless ratio, ζ. It can be shown from the figure that both λ and ζ increase with the increase in *Re*_*i*_ and *D* (curvature ratio), while *η*_*ex*_ decreases with increasing *Re*_*i*_ and *D* for all test specimens. Comparatively, with the *SSHTHX*, the heat transfer performance of *STHTHX* optimally outperforms at the same *D*. When *Re*_*i*_ rises from 7.57 × 10^3^ to 4.4 × 10^4^, the total increments in the λ of the *STHTHX* with *D* = 0.095 m, 0.125 m, and 0.165 m are 1794.3, 1159.3, and 759.6%, respectively. The increases in ζ are 237.8, 260.7, and 293.5%, respectively. While the decreases in *η*_*ex*_ are 9.9, 13.7, and 14.5%, respectively.

Furthermore, as* D* decreases from 0.165 m to 0.095 m the average decrements in λ and ζ are 25.9 and 35.1%, respectively. While *η*_*ex*_ rises by 10.4%. Comparatively, with the *SSHTHX,* the heat transfer performance of *STHTHX* optimally outperforms at the same *D.* The average decrements in λ and ζ are 13.1 and 16.7%, respectively. While *η*_*ex.*_ rises by 8.4%. This can describe the benefits of the twisted helical tube in *STHTHX* in decreasing the heat loss between the hot and cold fluids. When compared to the *SSHTHX*, the *STHTHX* has a higher heat transmission rate. This can be related to heat transmission in the *STHTHX*, the intensity level of the swirl flow, the boundary layer disrupting the flow, and the extra turbulence level of the flow caused by the tube twisting. As a result, irreversibility and energy losses also decline.

## Correlations

**Fig. 9:**
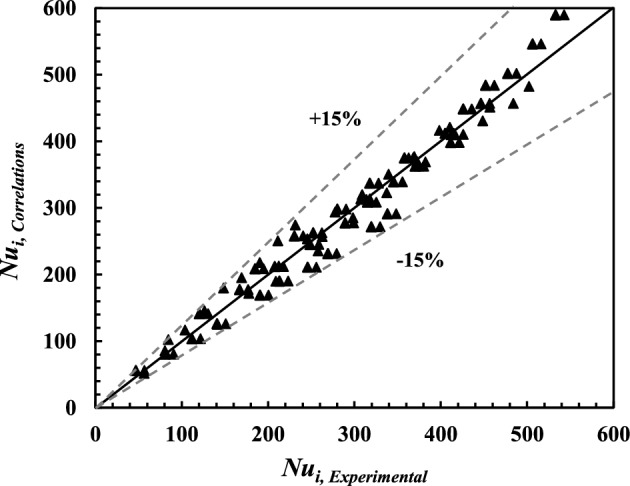
Verification of *Nu*_*i*_ for the present correlation and the experimental data.

**Fig. 10:**
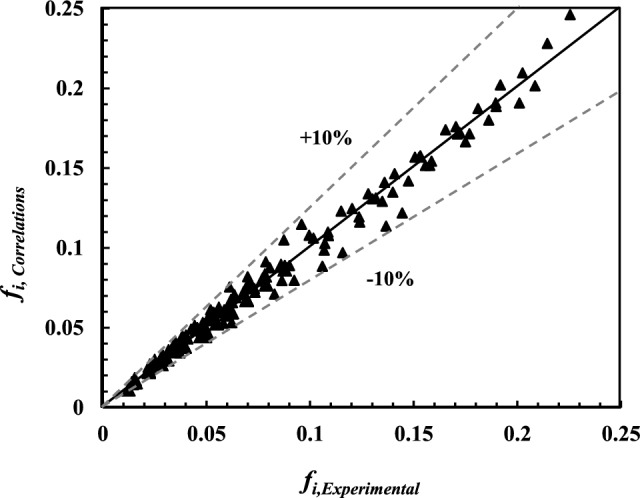
Verification of *f*_*i*_ for the present correlation and the experimental data.

The present experimental data was employed to develop new correlations to estimate both $\overline{{Nu_{i} }}$ and *f*_*i*_ for the twisted helical coil side of the *STHTHX.* A regression analysis method for $\overline{{Nu_{i} }}$ Eq. (25) and *f*_*i*_ Eq. (26) with deviations of ± 15% and ± 10%, respectively, as depicted in Figs. 9 and 10. The new correlations are valid for 6 × 10^3^ ≤ *Re*_*i*_ ≤ 4.4 × 10^3^, 3.6 ≤ *Pr*_*i*_ ≤ 4.14, and 0.056 ≤ *δ*_*i*_ ≤ 0.098 as:

25 $$\overline{{Nu_{i} }} = 0.0316Re_{i}^{1.102} Pr_{i}^{ - 1.43} \delta_{i}^{0.095}$$

26 $$f_{i} = 1.034e^{ - 3} Re_{i}^{0.502} Pr_{i}^{ - 2.41} \delta_{i}^{0.68}$$

## Conclusions

This work experimentally develops a new design of twisted helical tube in *STHTHX* under turbulent flow conditions and examines the thermo-fluid characteristics in comparison to *SSHTHX*. The impacts of twisted helical coil diameter, *Re*_*i*_, *Re*_*sh*_*.* on the thermal performance and exergy analysis of the *STHTHX* are analyzed by using *Nu*_*i*_, *f*_*i*_, *U*_*o*_*, η*, *λ*, *η*_*ex*_*,* and *ζ*. Furthermore, correlations for estimating and *f*_*i*_ are proposed. The study can expect to fill the research gap on the thermo-fluid characteristics and exergy analysis of the *STHTHX*; this work can also provide experimental guidance for *STHTHX* design and optimization. The following are the key conclusions.

- The twisted helical tube heat exchanger (*STHTHX*) demonstrated superior thermofluid performance compared to the conventional smooth helical tube heat exchanger (*SSHTHX*).
- At the same Reynolds number (*Re*_*i*_ = 31,000), increasing the shell-side mass flow rate from 0.067 to 0.175 kg/s enhanced the performance by 3.87 times.
- The *U*_*o*_ of *STHTHX* presents an optimal performance compared to that of *SSHTHX*, and the *STHTHX* present an average grow in* U*_*o*_ by 33.7% compared to *SSHTHX*.
- The average *f*_*i*_ of *STHTHX* remained 84.1% higher than that of *SSHTHX*.
- Reducing the twisted coil diameter from 0.165 to 0.095 m increased $\overline{{Nu_{i} }}$ and *f*_*i*_ by 47.9% and 38.77%, respectively.
- The highest thermal performance (*η* = 2.07) was recorded for *D* = 0.095 m, followed by 1.82 and 1.54 for *D* = 0.125 and 0.165 m, respectively.
- As *D* decreased, the average decreases in *λ* and *ζ* were 25.9 and 35.1%, while *η*_*ex*_ increased by 10.4%.
- Compared with *SSHTHX*, *STHTHX* achieved lower *λ* and *ζ* values by 13.1 and 16.7%, respectively, and higher *η*_*ex*_ by 8.4%.

## Data Availability

The corresponding author will be made the data available upon request.

## List of symbols

*A*: Area, m^2^
*C*: Specific heat, J/kg.C
*d*: Diameter, m
*E*: Exergy, W
*f*: Friction factor
$\overline{{h_{i} }}$: Average convective heat transfer coefficient, W/m^2^.^o^C
*k*: Thermal conductivity, W = m C
*L*: Length, m
$\dot{m}$: Mass flow rate, kg/s
*N*: The twisted helical coil number of turns
$\overline{{Nu_{i} }}$: Average Nusselt number
*p*: Twisted height, m
*P*: Pressure, Pa
*Pr*: Prandtl number (*μC/k*)
*Q*: Heat transfer rate, W
*Re*: Reynolds number
s: Twisted pitch, m
*T*: Temperature, ^o^C
*U*_o_: Overall heat transfer coefficient, W/m^2^.^o^C
*v*: Mean axial velocity, m/s
*V*: Volume, m^3^

## Greek symbols

*δ*: Dimensionless twisted helical coil curvature ratio, (*d*_*t,i*_*/d*_*c*_)
*μ*: Dynamic viscosity, kg/m.s
*ρ*: Density, kg = m^3^
*ω*: Uncertainty
*λ*: Exergy destruction rate, W
*η*: Thermal performance index
*ζ*: Exergy dimensionless ratio

## Subscripts and superscripts

*avg*: Average
*c*: Coil
*ex.*: Exergy
*f*: Film/fluid
*h*: Hydraulic
*i*: Inner/inlet/internal
*LMTD*: Logarithmic mean temperature difference, ^o^C
*m*: Mean
*min*: Minimum
*o*: Out/outer
*s*: Surface
*S*: Straight tube
*sh*: Shell
*t*: Tube

## Abbreviations

*NTU*: Number of transfer unit
*STHTHX*: Shell and twisted helical tube heat exchanger
*SSHTHX*: Shell and smooth helical tube heat exchanger
